# Insulin Dependence Increases the Risk of Complications and Death in Total Joint Arthroplasty: A Systematic Review and Meta‐(Regression) Analysis

**DOI:** 10.1111/os.12944

**Published:** 2021-03-18

**Authors:** Li‐min Wu, Hai‐bo Si, Ming‐yang Li, Yuan‐gang Wu, Yi Zeng, Bin Shen

**Affiliations:** ^1^ Department of Orthopaedics, Orthopedic Research Institute, West China Hospital Sichuan University Chengdu China; ^2^ Department of Orthopaedics and National Clinical Research Center for Geriatrics, Orthopedic Research Institute, West China Hospital Sichuan University Chengdu China

**Keywords:** Complications, Diabetes Mellitus, Insulin, Prevalence, Total joint arthroplasty

## Abstract

**Objectives:**

To investigate the proportion of insulin‐dependent diabetes mellitus (IDDM) patients among diabetic patients undergoing total joint arthroplasty (TJA) and whether insulin dependence is associated with postoperative complications.

**Methods:**

A systematic literature search was performed in EMBASE, PubMed, Ovid, Medline, the Cochrane Library, Web of Science, the China Science and Technology Journal Database, and China National Knowledge Infrastructure from the inception dates to 10 September 2019. Observational studies reporting adverse events with IDDM following TJA were included. Primary outcomes were cardiovascular complications, pulmonary complications, kidney complications, wound complications, infection, and other complications within 30 days of surgery. Secondary outcomes were the proportion of IDDM patients among diabetic patients undergoing TJA and its time trend.

**Results:**

A total of 19 studies involving 85,689 participants were included. Among patients undergoing TJA, 26% of diabetic patients had IDDM. Compared with non‐insulin‐dependent diabetes (NIDDM), the incidences of cardiac arrest (risk ratio [*RR*], 2.346; 95% confidence interval [*CI*], 1.553 to 3.546), renal failure (relative risk [*RR*], 2.758; 95% *CI*, 1.830 to 4.156), deep incisional surgical site infection (*RR*, 1.968; 95% *CI*, 1.107 to 3.533), wound dehiscence (*RR*, 2.209; 95% *CI*, 1.830 to 4.156), and death (*RR*, 2.292; 95% *CI*, 1.568 to 3.349) were all significantly increased in IDDM. A significant time trend was witnessed for the prevalence of IDDM (*P* = 0.014). There was no statistical significance for organ/space surgical site infection, thrombotic events (deep venous thrombosis/ pulmonary embolism), and revision rates.

**Conclusion:**

Insulin‐dependent diabetes is an independent high‐risk factor for increased adverse outcomes relative to NIDDM, suggesting that hierarchical and optimal blood glucose management may contribute to reducing the adverse complications after surgery for these patients. In addition, because the risk of sepsis, deep wound infection, organ/space surgical site infection, urinary tract infection, renal insufficiency, and renal failure significantly increase after TJA in IDDM patients, more active postoperative antimicrobial prophylaxis may be needed on the premise of protecting renal function.

## Graphical abstract

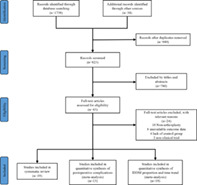

## Introduction

Total joint replacement (TJA) is a common and effective method to relieve the pain from osteoarthritis (OA). Globally, the rate of TJA is projected to increase accordingly with the rising prevalence of OA^1^, ^2^, ^3^. Meanwhile, diabetes mellitus (DM) is a pressing public health issue, and its prevalence is expected to increase by 69% and 20%, respectively, in developing and developed countries between 2010 and 2030^4^. There is evidence from clinical and animal models to suggest an underlyingly independent link between DM and severity of OA^5^. Therefore, the number of TJA procedures is likely to increase in diabetic patients. Furthermore, an association between DM and adverse events, hospital readmission, and increased death rates has been witnessed in lower extremity arthroplasty and shoulder arthroplasty^6^, ^7^.

However, these aforementioned studies generally classify patients as DM and non‐DM patients, without further considering whether they are relying on insulin to achieve glycemic control. Studies have found that patients dependent on insulin have a higher risk of perioperative adverse events, especially cardiac complications after elective non‐cardiac surgery^8^, ^9^. Although this concept of stratification has recently been reported in TJA patients^10^, the relationship between insulin dependence and risk of adverse events has not been well recognized. This information will help in managing patients’ expectations, in preoperative risk stratification, in implementing appropriate prevention, and with monitoring measures.

This meta‐analysis aims to answer the following questions. First, what is the current proportion of insulin‐dependent DM (IDDM) in diabetic patients undergoing TJA? Second, has there been an increase in the proportion of IDDM in TJA over the past decade? Finally, is IDDM associated with an increased risk of adverse events after TJA.

## Method

This meta‐analysis was performed in accordance with the *Cochrane Handbook for Systematic Reviews of Interventions* and the PRISMA Checklist guidelines^11^, ^12^ (Appendix 2).

### Search Strategy

The electronic databases of EMBASE, PubMed, Ovid, Medline, the Cochrane Library, Web of Science, China Science and Technology Journal Database, and China National Knowledge Infrastructure were searched from the inception dates to 10 September 2019. The search terms were as follows: (Human Isophane Insulin OR Protophane OR Protophan OR Insulin OR Humulin OR Novolin OR Insularaed) AND (arthroplasty OR knee replacement OR shoulder replacement OR hip replacement). We also identify other possible original studies by searching the Google search engine.

### Inclusion Criteria

Studies were included based on the following criteria: (i) cohort or case‐control studies focused on the influence of IDDM on the postoperative complication rate of total joint arthroplasty (TJA); (ii) sufficient sample size in each study; (iii) availability of reported outcomes, including proportion of IDDM patients, cardiac arrest, stroke, sepsis, myocardial infarction, extended length of stay (>5 days), on ventilator >48 h, renal failure, superficial incisional surgical site infection (SSI), deep incisional SSI, organ/space SSI, thrombotic event (vein thrombosis embolism/pulmonary embolism), reoperation, readmission, wound dehiscence, urinary tract infection, renal insufficiency, revision, unplanned intubation, pneumonia, and death. The exclusion criteria were: (i) studies that used treatments involving glucose control or combining other drugs; (ii) studies enrolling patients undergoing unicompartmental joint replacement or acute joint arthroplasty due to fracture; and (iii) review articles, expert opinions, and trials that do not consider complications.

### Quality Assessment

Two authors (WLM and LMY) independently assessed the methodological quality of observational studies based on the nine‐star system of the Newcastle–Ottawa scale (NOS). The NOS evaluates study population representativeness, comparability of IDDM and non‐insulin dependent DM (NIDDM), assessment of outcomes, follow‐up length, and adequacy of follow up (Table 1).

**TABLE 1:** Characteristics of included studies

Authors/Year	Area	Surgery	Number of patients		Gender (F/M)		NOS	Evidence level
			IDDM	NIDDM	IDDM	NIDDM		
Lung 2019^13^	USA	TSA	380	922	207/173	494/428	7	III
Lee 2019^14^	USA	rTKA	1,171	2,335	626/544	1,325/1,010	7	III
Gu 2018^15^	USA	rTKA	975	1,890	516/459	1,065/825	6	III
Pelle 2018^16^	DK	TKA, THA	300	1,222	NA	NA	7	II
Webb 2017^17^	USA	TKA	4,881	15,367	2,775/2,106	9,210/6,157	7	III
Fu 2017^10^	USA	TSA	295	691	166/129	372/319	7	III
Webb 2016^18^	USA	TKA, THA	6,801	21,672	3,709/3,182	12,406/9,266	7	III
Bohl 2016^19^	USA	TKA, THA	4,298	12,297	NA	NA	5	II
Watts 2015^20^	USA	TKA	164	366	108/56	400/130	6	III
Gregory 2015^21^	USA	TSA	30	87	NA	NA	6	II
Lovecchio 2014^22^	USA	TKA, THA	1,552	5,173	878/674	3,016/2,157	6	III
Christoffer 2014^23^	DK	TKA, THA	42	890	NA	NA	6	II
Han 2013^24^	KR	TKA	32	135	NA	NA	6	III
Iorio 2012^25^	USA	TKA, THA	72	278	NA	NA	6	III
Jamsen 2012^26^	FIN	TKA, THA	245	629	NA	NA	6	II
Meding 2003^27^	FRA	TKA	118	211	NA	NA	5	II
Papagelopoulos 1996^28^	USA	TKA	12	39	NA	NA	6	III
Serna 1994^29^	USA	TKA	8	40	7/1	33/12	5	III
Bruce 1993^30^	USA	THA	17	52	NA	NA	5	III

Case‐control, case‐control study; F/M, female/male; IDDM, insulin‐dependent diabetes mellitus; rTKA, revision total knee arthroplasty; NA, not available; NIDDM, non‐insulin‐dependent diabetes mellitus; NOS, Newcastle‐Ottawa Scale; SD, mean difference; THA, total hip arthroplasty; TKA, total knee arthroplasty; TSA, total shoulder arthroplasty.

The evidence quality of outcomes was systematically assessed by the two reviewers (WLM and YP) based on the Grading of Recommendations Assessment, Development and Evaluation (GRADE) approach (https://gdt.gradepro.org/app/)^31^ (Appendix 1). Although the GRADE approach considers the results of observational studies as evidence of low quality, there are a series of criteria to improve the assessment of quality levels, including a substantial effect (risk increased or decreased at least twofold), a dose‐response gradient, no confounding factors, and no bias. The evidence quality of each outcome is shown in Table 4.

### Data Extraction

Data were independently extracted by two authors (WLM and LMY), including: author, year, area, design, surgery, sample size, age, gender, and rate of cardiac arrest, stroke, sepsis, myocardial infarction, extended length of stay (>5 days), on ventilator >48 h, renal failure, superficial incisional SSI, deep incisional SSI, organ/space SSI, thrombotic event (vein thrombosis embolism/pulmonary embolism), reoperation, readmission, wound dehiscence, urinary tract infection, renal insufficiency, revision, unplanned intubation, pneumonia, and death. Disagreements were resolved by discussion. We also attempted to contact study authors by email for additional information if needed.

### Definition of Outcomes

The primary outcomes were cardiovascular complications, pulmonary complications, kidney complications, wound complications, infection, and other complications within 30 days of surgery (unless specifically mentioned). Understanding these outcomes can help in managing postoperative care and monitoring, improving the quality and safety of TJA. Secondary outcomes were the proportion of IDDM patients among diabetic patients undergoing TJA and its time trend.

### Cardiovascular Complications

Cardiac arrest (requiring external or open cardiopulmonary resuscitation), stroke (resulting in residual neurologic deficit), and myocardial infarction (defined on the presence of the ICD‐9‐CM code 410.xx) were considered cardiovascular complications.

### Pulmonary Complications

On ventilator >48 h, unplanned intubation, and pneumonia (hospitalized or radiologically confirmed) were considered pulmonary complications.

### Kidney Complications

Renal insufficiency (an increase in serum creatinine level ≥0.3 mg/dL within 48 h) and renal failure (deterioration in renal function sufficient to require dialysis) were considered kidney complications.

### Wound Complications

Wound dehiscence, superficial incisional SSI (infections with purulent drainage that occurred at the incision sites), and deep incisional SSI (clinically diagnosed infections below the fascia or joint capsule with persistent wound discharge or joint pain) were considered wound complications.

### Infection

Urinary tract infections (an infection in the kidneys, ureters, bladder, or urethra), organ/space SS (infection involves organs or spaces), and sepsis (severe infection resulting in multiple organ affection) were considered postoperative infections.

### Other Complications

Extended length of stay (>5 days), reoperation (unplanned return to the operating room or procedure requiring a second anesthetic event), thrombotic events (VTE and PE within 90 days), readmission (≥1 night in hospital and potentially surgically related), prosthetics revision (removal or replacement of at least one prosthetic component), and death were considered other complications.

### Data Analysis

The relative risk (*RR*) and 95% confidence interval (*CI*) were calculated in Stata/SE (15.1 for Mac 64‐bit Intel) for discontinuous data; *P* < 0.05 was considered statistical significance. The *I* ^2^ statistic was used for calculating the heterogeneity of eligible studies and its results were interpreted as follows: 25%, low heterogeneity; 50%, moderate heterogeneity; and 75%, high heterogeneity. To address heterogeneity and publication bias, multiple sensitivity analyses, including outlier removal, subgroup analysis according to type of surgery and areas, Duval and Tweedie's trim, and fill methods, were performed. If *I* ^2^ still existed (>50%), we used the random‐effect model. Egger and Harbord's test based on outcomes was used to assess publication bias for continuous and discontinuous data, respectively.

Moment mixed‐effect meta‐regression was applied to assess time trends, and due to the varied duration of study enrollment, the beginning, middle, and end year of patient enrollment were used for regression.

## Results

### Search Results

**Fig. 1:**
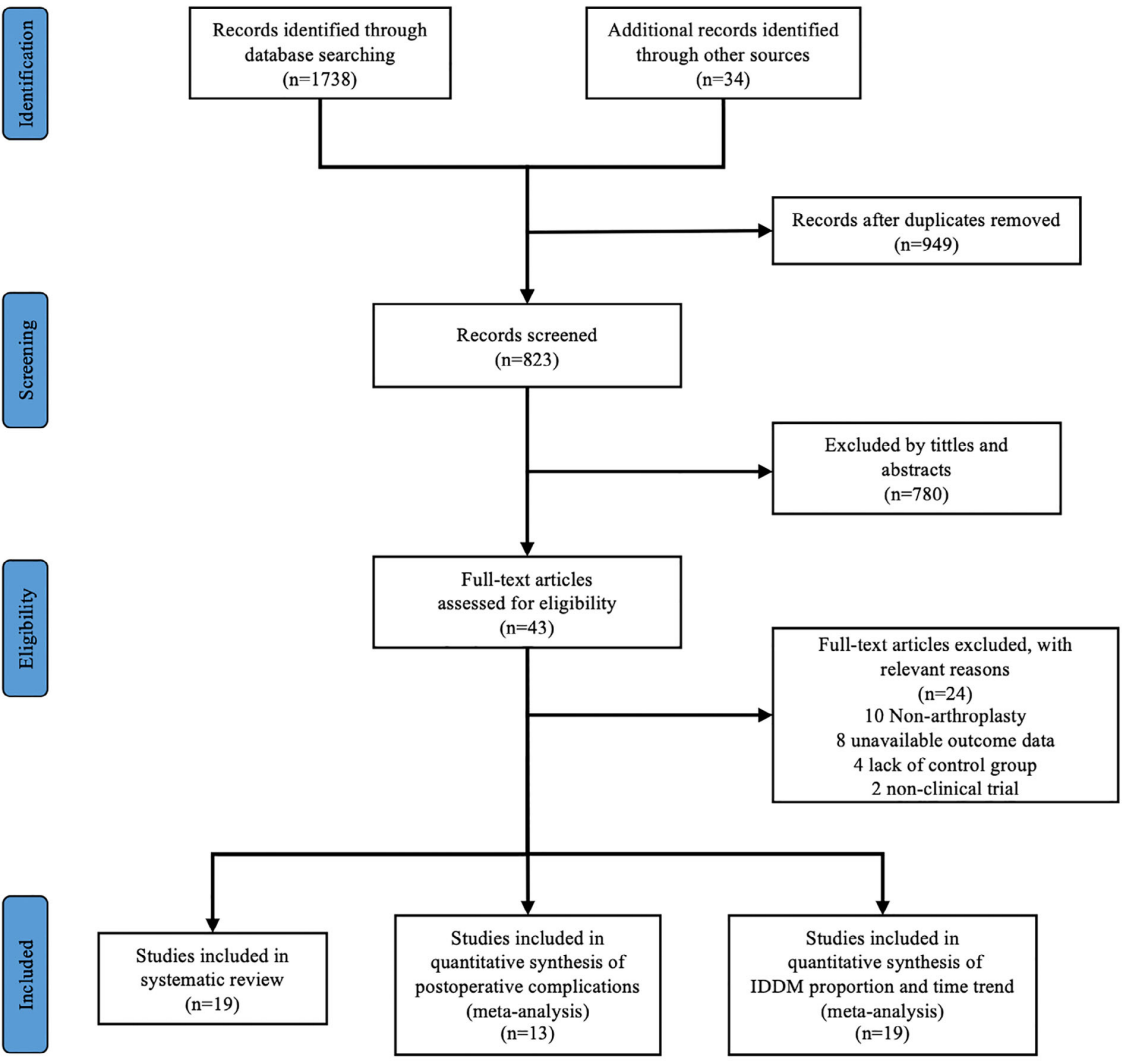
Study selection.

There were 1772 initial studies obtained after systematic searching. A total of 949 duplicates and 780 articles were excluded by screening abstracts. We ended up with 43 eligible studies: 19 met the inclusion criteria, 10 studies were not on TJA, 8 studies did not contain available data, 4 articles did not contain a control group, and 2 studies were non‐clinical trials and were excluded. The flow chart in Fig. 1 shows the selection process of eligible studies.

### Study Characteristics

The included 19 studies were published between June 1993 and July 2019, involving 85,689 patients undergoing TJA. The included articles’ demographics are listed in Table 1. The specific complications developed are further listed in Tables 2 and 3. All but 14 studies were conducted in the United States^10^, ^13^, ^14^, ^17^, ^18^, ^19^, ^20^, ^21^, ^22^, ^25^, ^28^, ^29^, ^30^; 2 were from Denmark^16^, ^23^, and the remaining 3 articles were from France^27^, Finland^26^, and Korea^24^, respectively. The mean age of patients among the groups ranged from 63 to 70.1 years. In this meta‐analysis, 3 articles included participants undergoing total shoulder arthroplasty (TSA)^10^, ^13^, ^21^, 6 included total knee arthroplasty (TKA)^17^, ^20^, ^24^, ^27^, ^28^, ^29^, 2 included revision total knee arthroplasty (rTKA)^14^, ^15^, 1 included total hip arthroplasty (THA)^30^, and the rest of the studies included both TKA and THA^16^, ^18^, ^19^, ^22^, ^23^, ^25^, ^26^. The mean NOS score was 6.18 (maximum 9), suggesting that this meta‐analysis included high‐quality studies.

**TABLE 2:** Main clinical outcomes of included studies

Study	Design	Period	Outcome assessment	Outcomes determined	Adjusted factors
Lung^13^	Case–control	2015–2016	Readmission Non‐routine discharge Postoperative SSI Postoperative renal failure Postoperative myocardial infarction Postoperative pneumonia Postoperative PE Postoperative transfusion due to bleeding	None	Baseline characteristics
Lee^14^	Retrospective	2005–2016	Superficial incisional SSI Deep incisional SSI Organ/space SSI Wound disruption Pneumonia Unplanned Intubation Pulmonary Embolism Ventilator Dependence (>48 h) Renal insufficiency Acute renal failure Urinary tract Infection Stroke Cardiac arrest Myocardial Infarction Blood transfusions Deep venous Thromboembolism (DVT) systemic sepsis Septic shock Death Return to operating Room extended Length of stay (>5 days) Readmission	Pneumonia Blood transfusions Septic shock Extended LOS (>5 days)	Unadjusted
Gu^15^	Retrospective	2007–2016	Death Cardiac complications Acute renal failure Renal insufficiency Pulmonary complications VTE/PE Stroke Sepsis Deep SSI Superficial SSI Wound dehiscence Urinary tract infection Return to operating room Extended LOS	Pneumonia Re‐intubate Fail to wean >48 h Renal fail Cardiac arrest Death Return to operating room Extended LOS Superficial SSI Postoperative blood transfusion	Unadjusted
Pelle^16^	Prospective	2010–2015	Myocardial infarction	Myocardial infarction	Unadjusted
Webb^17^	Retrospective	2005–2014	Death Cardiac arrest Stroke Sepsis Myocardial infarction Renal failure Thrombotic event (PE/DVT) wound‐related infection On ventilator >48 h Unplanned intubation Renal insufficiency Return to the operating room Wound dehiscence Readmission Pneumonia Urinary tract infection Extended LOS (>5 days)	Sepsis Myocardial infarction Renal failure On ventilator >48 h Unplanned intubation Renal insufficiency Return to the operating room Wound dehiscence Readmission Pneumonia Urinary tract infection Extended LOS (>5 days)	Age Gender Age CCI Smoking status
Fu^10^	Retrospective	2011–2014	Any complications Cardiac arrest Myocardial infarction Pulmonary complications Renal insufficiency, Stroke VTE PE Sepsis Reoperation Death Wound complications Urinary tract infection Blood transfusion Readmission Extended LOS.	Any complications Stroke Blood transfusion Extended LOS	Gender Age BMI Modified CCI Hypertension, Cardiac history COPD Current smoking Preoperative functional status
Webb^18^	Retrospective	2005–2014	Death Cardiac arrest Stroke Sepsis Myocardial infarction Renal failure Thrombotic event (PE/DVT) Wound‐related infection On ventilator >48 h Unplanned intubation Renal insufficiency Return to the operating room Wound dehiscence Readmission Pneumonia Urinary tract infection Extended length of stay (>5 days)	Sepsis Myocardial infarction Renal failure Thrombotic event (PE/DVT) On ventilator >48 hours Unplanned intubation Renal insufficiency Return to the operating room Wound dehiscence Readmission Pneumonia Urinary tract infection Extended length of stay (>5 days)	Age Gender Age CCI Smoking status
Bohl^19^	Prospective	2005–2013	Sepsis	Sepsis	Baseline demographic, Comorbidity
Watts^20^	Retrospective	1995–2011	Reoperation Revision Periprosthetic Joint Infection	Reoperation Revision Periprosthetic Joint Infection	Unadjusted
Gregory^21^	Prospective	2005–2012	TSA Complication	TSA Complication	Unadjusted
Lovecchio^22^	Retrospective	2005–2011	Overall complication Surgical complications Superficial wound Deep incision/organ space Medical complications Pneumonia Unplanned intubation On ventilation >48 hours Renal insufficiency Urinary tract infection Stroke Cardiac arrest Myocardial infarction Sepsis Return to operating room Readmission 30‐day mortality rate	Overall complication Medical complications Pneumonia Unplanned intubation Renal insufficiency Urinary tract infectionStroke Myocardial infarction Sepsis Readmission 30‐day mortality rate	Age Sex Race BMI Smoking Steroid use Hypertension, COPD Anesthesia type
Christoffer^23^	Prospective	2010–2012	Extended LOS (>4 days) 30‐day readmissions 90‐day readmissions Diabetes‐related morbidity	Diabetes‐related morbidity	Demographics, Comorbidity Department of surgery
Iorio^25^	Retrospective	2004–2009	Infection	Infection	Unadjusted
Meding^27^	Prospective	1980–1989	Revision Deep infection Wound Neuropathy Manipulation Urinary tract infection	Deep infection Neuropathy Manipulation Urinary tract infection	Unadjusted
Serna^29^	Retrospective	1987–1999	Revision	None	Unadjusted

BMI, body mass index; CCI, Charlson comorbidity index; COPD, chronic obstructive pulmonary disease; DVT, deep vein thrombosis; LOS, length of stay; PE, pulmonary embolism; SSI, surgical site infection; TSA, total shoulder arthroplasty; VTE, venous thromboembolism.

**TABLE 3:** Specific incidence of postoperative complications for eligible studies (%)

Author	Status	Any	CR	Stroke	Sepsis	MI	LOS(>5d)	Ventilator>48 h	RF/RI	SupSSI	Deep SSI	O/S SSI	VTE/PE	Re‐op	Re‐ad	Wound dehiscence	Urinary tract infection	Revision	Pneumonia	Death
Lung ^13^	IDDM	NA	NA	NA	NA	0.5%	NA	NA	0%	NA	NA	NA	NA	NA	5.5%	NA	NA	NA	0.5%	NA
	NIDDM	NA	NA	NA	NA	0.1%	NA	NA	0.1%	NA	NA	NA	NA	NA	4.2%	NA	NA	NA	0.8%	NA
Lee^14^	IDDM	NA	0.5%	0.2%	2.6%	0.6%	23%	0.3%	0.9%	0.4%	1.0%	1.6%	1.1%	4.5%	6.6%	1%	1.3%	NA	2%	0.6%
	NIDDM	NA	0.0%	0.2%	1.6%	0.4%	17%	0.0%	0.6%	1.0%	0.4%	1.8%	1.3%	3.5%	6.1%	1%	1.5%	NA	0%	0.3%
Gu^15^	IDDM	31.0%	0.4%	0.1%	2.3%	0.5%	NA	0.5%	1.4%	0.2%	1.1%	0.5%	1.0%	4.5%	NA	0.5%	1.1%	NA	1.3%	0.6%
	NIDDM	21.7%	0.0%	0.1%	1.5%	0.3%	NA	0.0%	0.5%	0.8%	0.6%	0.4%	1.3%	3.0%	NA	0.4%	1.3%	NA	0.4%	0.0%
Pelle^16^	IDDM	NA	NA	NA	NA	1.0%	NA	NA	NA	NA	NA	NA	NA	NA		NA	NA	NA	NA	NA
	NIDDM	NA	NA	NA	NA	0.2%	NA	NA	NA	NA	NA	NA	NA	NA		NA	NA	NA	NA	NA
Webb ^17^	IDDM	NA	0.2%	0.2%	0.7%	0.5%	9.0%	0.2%	0.8%	NA	NA	NA	1.5%	1.9%	5.0%	0.5%	1.5%	NA	0.8%	0.1%
	NIDDM	NA	0.1%	0.1%	0.4%	0.3%	6.4%	0.1%	0.3%	NA	NA	NA	1.6%	1.3%	3.3%	0.2%	1.1%	NA	0.4%	0.1%
Fu^10^	IDDM	14.6%	NA	0.7%	0.7%	NA	NA	NA	NA	NA	NA	NA	0.7%	2.0%	4.0%	NA	1.7%	NA	NA	0.7%
	NIDDM	9.3%	NA	0.0%	0.0%	NA	NA	NA	NA	NA	NA	NA	0.7%	0.9%	2.9%	NA	0.4%	NA	NA	0.1%
Webb ^18^	IDDM	NA	0.2%	0.2%	0.9%	0.7%	9.7%	0.3%	0.8%	NA	NA	NA	1.3%	2.4%	5.7%	0.4%	1.7%	NA	0.4%	0.3%
	NIDDM	NA	0.1%	0.1%	0.5%	0.3%	6.5%	0.1%	0.3%	NA	NA	NA	1.3%	1.7%	3.7%	0.2%	1.1%	NA	0.4%	0.1%
Bohl^19^	IDDM	NA	NA	NA	1.0%	NA	NA	NA	NA	NA	NA	NA	NA	NA	NA	NA	NA	NA	NA	NA
	NIDDM	NA	NA	NA	0.4%	NA	NA	NA	NA	NA	NA	NA	NA	NA	NA	NA	NA	NA	NA	NA
Watts^20^	IDDM	NA	NA	NA	NA	NA	NA	NA	NA	NA	NA	NA	NA	20.1%	NA	NA	NA	9.1%	NA	NA
	NIDDM	NA	NA	NA	NA	NA	NA	NA	NA	NA	NA	NA	NA	8.5%	NA	NA	NA	1.6%	NA	NA
Lovecchio 22	IDDM	8.8%	0.3%	0.5%	1.4%	0.8%	NA	0.1%	0.6%	NA	NA	NA	NA	2.1%	8.0%	NA	2.4%	NA	0.9%	0.6%
	NIDDM	6.3%	0.2%	0.1%	0.8%	0.5%	NA	0.3%	0.3%	NA	NA	NA	NA	1.7%	4.9%	NA	1.9%	NA	0.4%	0.3%
Christoffer 23	IDDM	NA	NA	NA	NA	NA	NA	NA	NA	NA	NA	NA	NA	NA	16.7%	NA	NA	NA	NA	NA
	NIDDM	NA	NA	NA	NA	NA	NA	NA	NA	NA	NA	NA	NA	NA	10.2%	NA	NA	NA	NA	NA
Meding^27^	IDDM	NA	NA	NA	NA	NA	NA	NA	NA	NA	NA	NA	NA	NA	NA	NA	3.4%	4.2%	NA	NA
	NIDDM	NA	NA	NA	NA	NA	NA	NA	NA	NA	NA	NA	NA	NA	NA	NA	0%	3.3%	NA	NA
Serna^29^	IDDM	NA	NA	NA	NA	NA	NA	NA	NA	NA	NA	NA	NA	NA	NA	NA	NA	0.0%	NA	NA
	NIDDM	NA	NA	NA	NA	NA	NA	NA	NA	NA	NA	NA	NA	NA	NA	NA	NA	9.1%	NA	NA

Any, any complications; CR, cardiac arrest; Deep SSI, deep incisional surgical site infection; IDDM, insulin dependent diabetes mellitus; LOS(>5 days), extended length of stay >5 days; MI, myocardial infarction; NA, not available; NIDDM, non‐insulin dependent diabetes mellitus; O/S SSI, organ/space surgical site infection; Re‐ad, readmission; Re‐op, reoperation; RF/RI, renal failure/renal insufficiency; Sup SSI, superficial surgical site infection; VTE/PE, venous thromboembolism/pulmonary embolism.

### Proportion

**Fig. 2:**
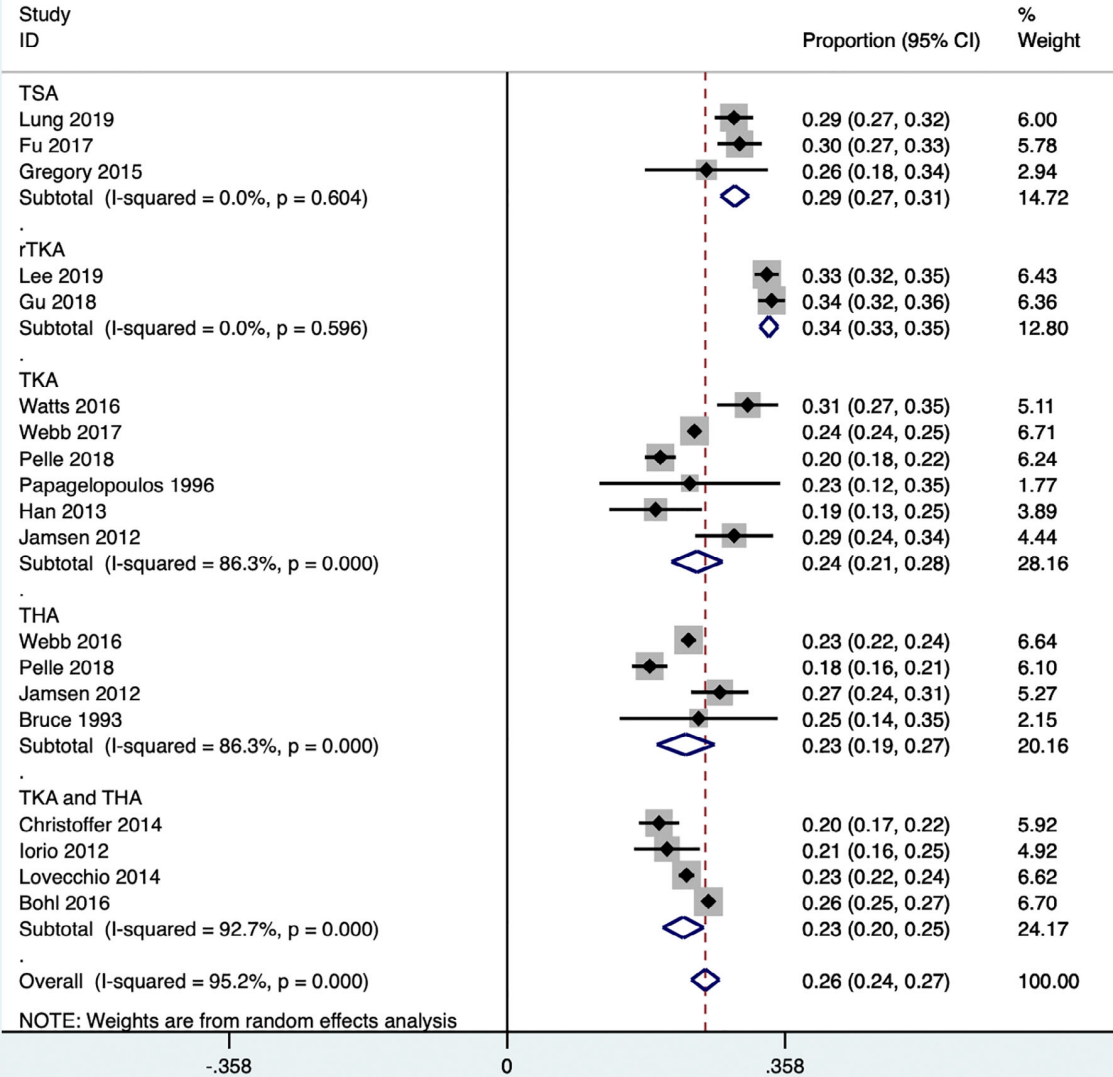
Proportion of insulin‐dependence in patients with diabetes mellitus (stratified by thetype of surgery)

**Fig. 3:**
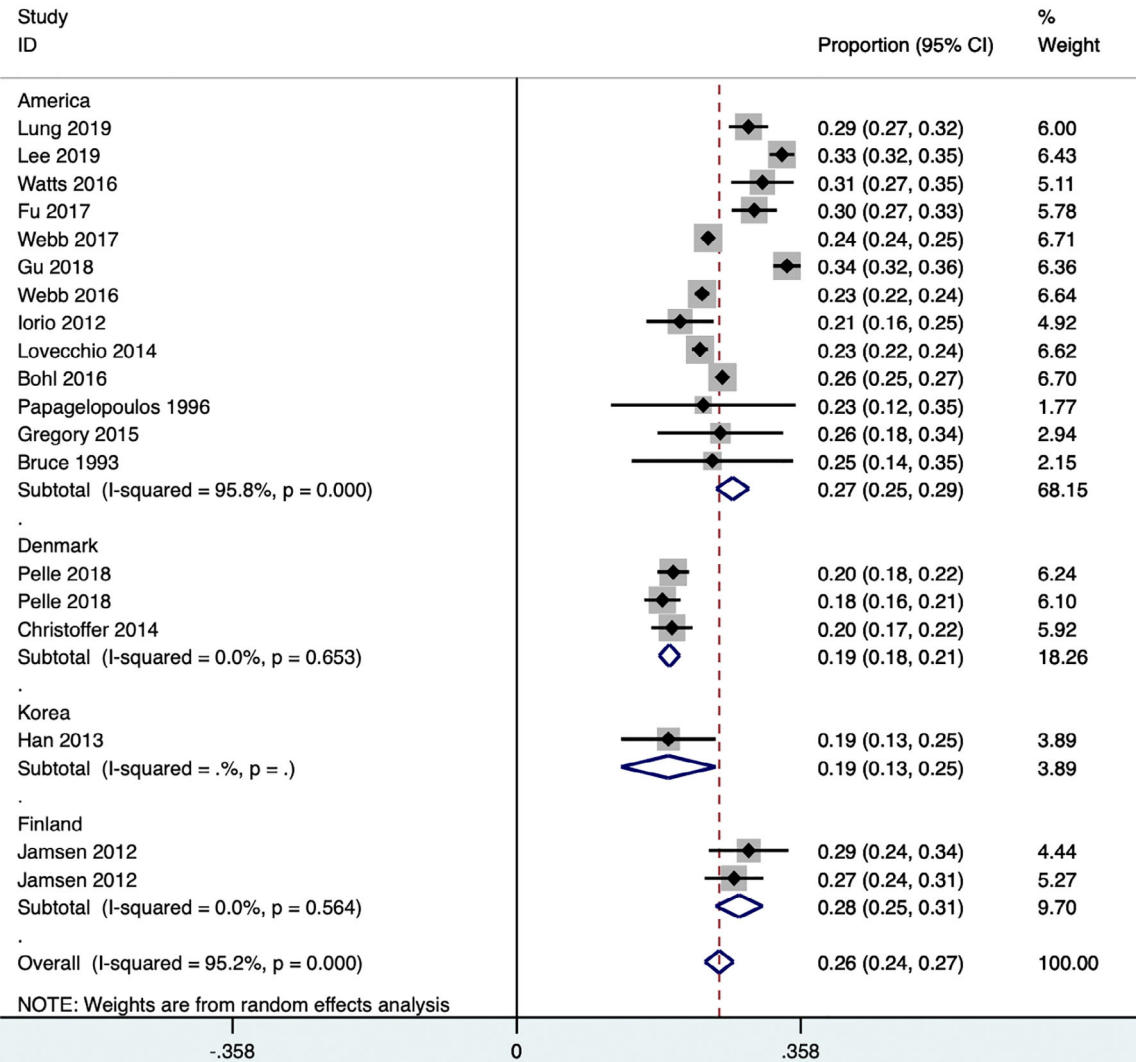
Proportion of insulin‐dependent in patients with diabetes mellitus (stratified by areas).

All 19 studies that were included reported the specific numbers of NIDDM and IDDM patients. Two articles^27^, ^29^ were excluded because there were too few participants and there was high heterogeneity in the sensitivity analysis. Two studies reported the proportion of IDDM patients in among TSA patients^10^, ^13^, ^21^, whereas 2 articles reported the proportion of IDDM patients among revision knee arthroplasty patients^14^, ^15^, 4 reported the proportion of IDDM patients among THA patients^16^, ^17^, ^26^, ^30^, 6 studies reported the proportion of IDDM patients among TKA patients^16^, ^18^, ^20^, ^24^, ^26^, ^28^ and 1 study separately reported the proportion of IDDM patients among THA and TKA patients^16^. In addition, 4 studies separately reported the proportion of IDDM patients among THA and TKA patients^19^, ^22^, ^23^, ^25^. The pooled analysis showed that the proportion of IDDM patients in total joint replacement group accounted for 26% (95% *CI*, 24% to 28%) of DM patients (Egger's test, *P* = 0.518) (Fig. 2). Because of the high heterogeneity of TKA, THA, and TKA and THA groups, we conducted the subgroup analysis according to the research areas. The results showed that the proportion of IDDM patients in the Denmark group was obviously lower than that in the United States (Fig. 3), which potentially explains the high heterogeneity.

### Time Trend in the Proportion of Patients with Insulin Dependence

**Fig. 4:**
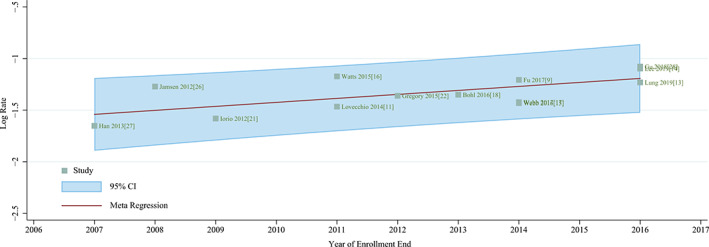
Meta‐regression for the prevalence of insulin‐dependent patients among diabetes mellitus patients.

Significant time trends in the proportion of patients with insulin dependence were not observed in the first and middle enrollment year for studies published after 2000 in the meta‐regression (*P* = 0.509 and 0.149, respectively). However, the meta‐regression showed a significant trend in the end enrollment year for studies published after 2000, with an outlier^16^ removed (*P* = 0.014) (Fig. 4).

### Postoperative Complications

The results of the meta‐analyses are listed in Table 4.

**TABLE 4:** Results of the meta‐analysis

Outcomes	Number of studies	Number of patients		RR (95% CI)	*P*	Heterogeneity	Model	Harbord's test	GRADE evidence
		IDDM	NIDDM						
Cardiac arrest	5^[^ ^7^, ^14^, ^20^, ^24^, ^31^ ^]^	15,380	46,437	2.343 (1.546 to 3.550)	***P* < 0.001**	*I* ^2^ = 0%, *P* = 0.529	Fixed	*P* = 0.160	⊕ ⊕ ⊕□
Stroke	6^[^ ^7^, ^9^, ^14^, ^20^, ^24^, ^31^ ^]^	15,675	47,128	2.180 (1.434 to 3.313)	***P* < 0.001**	*I* ^2^ = 6.6%, *P* = 0.374	Fixed	*P* = 0.649	⊕ ⊕ ⊕□
Sepsis	7^[^ ^7^, ^9^, ^14^, ^17^, ^20^, ^24^, ^31^ ^]^	19,973	59,425	1.952 (1.643 to 2.319)	***P* < 0.001**	*I* ^2^ = 0%, *P* = 0.684	Fixed	*P* = 0.330	⊕ ⊕ □□
Myocardial infarction	7^[^ ^7^, ^14^, ^17^, ^20^, ^23^, ^24^, ^31^ ^]^	16,262	48,460	1.854 (1.442 to 2.382)	***P* < 0.001**	*I* ^2^ = 0%, *P* = 0.807	Fixed	*P* = 0.699	⊕ ⊕ □□
Extended length of stay (>5 days)	3^[^ ^7^, ^14^, ^20^ ^]^	12,853	39,374	1.448 (1.362 to 1.539)	***P* < 0.001**	*I* ^2^ = 0%, *P* = 0.744	Fixed	*P* = 0.693	⊕ ⊕ □□
On ventilator >48 h	5^[^ ^13^, ^14^, ^20^, ^24^, ^31^ ^]^	15,380	46,437	2.918 (1.912 to 4.453)	***P* < 0.001**	*I* ^2^ = 0%, *P* = 0.617	Fixed	***P* = 0.027**	⊕ ⊕ ⊕□
Renal failure	5^[^ ^13^, ^14^, ^20^, ^24^, ^29^ ^]^	14,208	42,186	2.753 (1.822 to 4.158)	***P* < 0.001**	*I* ^2^ = 0%, *P* = 0.522	Fixed	*P* = 0.990	⊕ ⊕ ⊕□
Superficial incisional SSI	2^[^ ^13^, ^24^ ^]^	2,146	4,225	0.354 (0.159 to 0.790)	***P* = 0.011**	*I* ^2^ = 0%, *P* = 0.515	Fixed	‐	⊕ ⊕ ⊕□
Deep incisional SSI	2^[^ ^13^, ^24^ ^]^	2,146	4,225	1.967 (1.106 to 3.498)	***P* = 0.021**	*I* ^2^ = 0%, *P* = 0.962	Fixed	‐	⊕ ⊕ □□
Organ/Space SSI	2^[^ ^13^, ^24^ ^]^	2,146	4,225	0.916 (0.608 to 1.380)	*P* = 0.676	*I* ^2^ = 0%, *P* = 0.822	Fixed	‐	⊕ ⊕ □□
Thrombotic event (VTE/PE)	5^[^ ^9^, ^13^, ^14^, ^20^, ^24^ ^]^	14,123	41,955	0.975 (0.829 to 1.148)	*P* = 0.764	*I* ^2^ = 0%, *P* = 0.970	Fixed	*P* = 0.177	⊕ ⊕ □□
Reoperation	7^[^ ^9^, ^13^, ^14^, ^18^, ^20^, ^24^, ^31^ ^]^	15,839	47,494	1.444 (1.286 to 1.622)	***P* < 0.001**	*I* ^2^ = 15.9%, *P* = 0.309	Fixed	*P* = 0.246	⊕ ⊕ □□
Readmission	7^[^ ^9^, ^13^, ^14^, ^20^, ^21^, ^29^, ^31^ ^]^	15,122	47,050	1.494 (1.381 to 1.615)	***P* < 0.001**	*I* ^2^ = 15.8%, *P* = 0.227	Fixed	*P* = 0.374	⊕ ⊕ □□
Wound dehiscence	4^[^ ^13^, ^14^, ^20^, ^24^ ^]^	13,828	41,264	2.203 (1.596 to 3.040)	***P* < 0.001**	*I* ^2^ = 38.7%, *P* = 0.180	Fixed	*P* **= 0.048**	⊕ ⊕ ⊕□
Urinary tract infection	7^[^ ^9^, ^13^, ^14^, ^16^, ^20^, ^24^, ^31^ ^]^	15,793	47,339	1.400 (1.211 to 1.619)	*P* **= 0.009**	*I* ^2^ = 40.3%, *P* = 0.123	Fixed	*P* = 0.617	⊕ ⊕ □□
Renal insufficiency	5^[^ ^9^, ^13^, ^14^, ^20^, ^24^ ^]^	14,123	41,955	1.971 (1.141 to 3.403)	***P* = 0.015**	*I* ^2^ = 52.5%, *P* = 0.078	Random	***P* = 0.049**	⊕□□□
Revision	3^[^ ^16^, ^18^, ^22^ ^]^	291	621	2.330 (0.681 to 7.974)	*P* = 0.178	*I* ^2^ = 57.4%, *P* = 0.096	Random	*P* = 0.300	⊕□□□
Unplanned intubation	4^[^ ^13^, ^14^, ^20^, ^31^ ^]^	14,405	44,547	1.043 (0.715 to 1.521)	***P* = 0.028**	*I* ^2^ = 58.0%, *P* = 0.067	Random	***P* = 0.035**	⊕□□□
Pneumonia	6^[^ ^13^, ^14^, ^20^, ^24^, ^29^, ^31^ ^]^	15,760	47,359	1.965 (1.238 to 3.120)	***P* = 0.004**	*I* ^2^ = 65.6%, *P* = 0.013	Random	*P* = 0.631	⊕□□□
Death	6^[^ ^9^, ^13^, ^14^, ^20^, ^24^, ^31^ ^]^	15,675	47,128	2.292 (1.568 to 3.349)	***P* < 0.001**	*I* ^2^ = 0%, *P* = 0.464	Fixed	*P* = 0.354	⊕ ⊕ ⊕□

*CI*, confidence interval; GRADE, Grading of Recommendations Assessment, Development and Evaluation; IDDM, insulin dependent diabetes mellitus; NIDDM, non‐insulin dependent diabetes mellitus; No. of studies; No. of patients, number of patients; PE, pulmonary embolism; RR, relative risk; SSI, surgical site infection; VTE, venous thromboembolism. *p* < 0.05 values have been bold.

### Cardiovascular Complications

The occurrence of cardiac arrest was reported in 5 studies including 61,817 patients; no heterogeneity was found among these studies (*I* ^2^ = 0%), and cardiac arrest occurred more often in IDDM patients with an RR of 2.346 (95% *CI* 1.553 to 3.546). Stroke after TJA was documented in 6 studies, including 62,803 patients with *I* ^2^ of 0%. Stroke occurred more often in IDDM patients with an RR of 2.182 (95% *CI* 1.432 to 3.325). A total of 7 studies examined the risk of myocardial infarction. After pooled analysis, the result showed that an increased risk of myocardial infarction was associated with IDDM (*RR*, 1.874; 95% *CI* 1.461 to 2.402; *I* ^2^ = 0%).

### Pulmonary Complications

Four studies were able to be pooled for the occurrence of being on a ventilator >48 h with no heterogeneity (*I* ^2^ = 0%), and the results indicated an almost threefold greater rate in IDDM patients (*RR*, 2.868; 95% *CI*, 1.829 to 4.496). Although there were 4 and 5 articles reporting on the occurrence of unplanned intubation and pneumonia, respectively, they could not be pooled in the fixed‐effect model because of high heterogeneity (*I* ^2^ = 58% and 65%, respectively). Outlier removal was performed in these indictors; however, the heterogeneity remained at a high level (*I* ^2^ > 50%). In an effort to elucidate the unexplainable high heterogeneity, a predefined subgroup analysis according to type of TJA was performed on unplanned intubation and pneumonia (Table 5), and the association was found to be significant in both subgroups: TKA ([*RR*, 1.885; 95% *CI*, 1.099 to 3.235] and [*RR*, 2.279; 95% *CI*, 1.519 to 3.421], respectively) and rTKA ([*RR*, 2.833; 95% *CI*, 1.081 to 7.424] and [*RR*, 3.284; 95% *CI*, 1.866 to 5.781], respectively). Duval and Tweedie's trim and fill methods were used to test the stability of the model and there was significant increased risk of pneumonia (*RR*, 1.759; 95% *CI*, 1.074 to 2.879; *P* = 0.025). No significant increased risk was witnessed with unplanned intubation (*RR*, 1.312; 95% *CI*, 0.837 to 2.058, *P* = 0.237). After pooling the results in the random‐effect model, the meta‐analysis both showed increased risks of unplanned intubation (*RR*, 1.043; 95% *CI*, 0.715 to 1.521) and pneumonia (*RR*, 1.759; 95% *CI*, 1.073 to 2.882) in IDDM patients.

**TABLE 5:** Results of the subgroup analyses

Outcomes	Subgroup	Number of studies	Number of patients		RR (95% CI)	*P*	Heterogeneity	Model
			IDDM	NIDDM				
Unplanned intubation								
	TKA	1^17^	4,860	15,332	1.885 (1.099 to 3.235)	***P* = 0.021**	‐	Random
	rTKA	1^14^	1,161	2,328	2.833 (1.081 to 7.424)	***P* = 0.034**	‐	Random
	TKA, THA	2^18^, ^22^	8,306	26,719	1.433 (0.674 to 3.045)	*P* = 0.350	*I* ^2^ = 69.2%, *P* = 0.071	Random
	*Overall*	4^14^, ^17^, ^18^, ^22^	14,405	44,547	1.697 (1.059 to 2.718)	***P* = 0.028**	*I* ^2^ = 58.0%, *P* = 0.067	Random
Pneumonia								
	TKA	1^17^	4,841	15,312	2.279 (1.519 to 3.421)	***P* < 0.001**	‐	Random
	rTKA	2^14^, ^15^	2,114	4,206	3.284 (1.866 to 5.781)	***P* < 0.001**	*I* ^2^ = 0.0%, *P* = 0.882	Fixed
	TSA	1^13^	378	915	0.695 (0.145 to 3.330)	*P* = 0.649	‐	Random
	TKA, THA	2^18^, ^22^	8,312	26,739	1.431 (0.668 to 3.067)	*P* = 0.357	*I* ^2^ = 72.8%, *P* = 0.055	Random
	*Overall*	6^13^, ^14^, ^15^, ^17^, ^18^, ^22^	15,760	47,359	1.965 (1.238 to 3.120)	***P* = 0.004**	*I* ^2^ = 65.6%, *P* = 0.013	Random
Renal insufficiency								
	TKA	1^17^	4,858	15,342	2.888 (1.641 to 5.082)	***P* < 0.001**	‐	Random
	rTKA	2^14^, ^15^	6,766	21,634	1.125 (0.446 to 2.839)	*P* = 0.802	*I* ^2^ = 37.2%, *P* = 0.207	Random
	TSA	1^10^	2,134	4,204	0.335 (0.017 to 6.574)	*P* = 0.470	‐	Random
	TKA, THA	1^18^	6.766	21,634	2.925 (1.850 to 4.626)	***P* < 0.001**	‐	Random
	*Overall*	5^10^, ^14^, ^15^, ^17^, ^18^	14,123	41,955	1.971 (1.141 to 3.403)	***P* = 0.015**	*I* ^2^ = 52.5%, *P* = 0.078	Random

*CI*, confidence interval; NIDDM, non‐insulin dependent diabetes mellitus; No. of studies; No. of patients, number of patients; IDDM, insulin dependent diabetes mellitus; rTKA, revision total knee arthroplasty; RR, relative risk; THA, total hip arthroplasty; TKA, total knee arthroplasty; TSA, total shoulder arthroplasty. *P* < 0.05 values have been bold.

### Kidney Complications

Adverse events that occurred in kidneys were separately reported as renal failure and renal insufficiency in 5 studies with *I* ^2^ of 0% and 52.5%, respectively. After pooling the results in the fixed and random‐effect model, respectively, the results both indicated a twofold greater rate of adverse events in IDDM patients ([*RR*, 2.758; 95% *CI*, 1.830 to 4.156] and [*RR*, 1.976; 95% *CI*, 1.142 to 3.418] respectively). Due to high heterogeneity, the predefined subgroup analysis (stratified by the type of TJA) was performed for renal insufficiency (Table 5), and the association was significant in two subgroups: TKA and TKA/THA ([*RR*, 2.888; 95% *CI*, 1.641 to 5.082] and [*RR*, 2.925; 95% *CI*, 1.850 to 4.626], respectively). Duval and Tweedie's trim and fill methods were further performed to test the model stability and the results still showed positive significance (*RR* = 1.981, 95% *CI*: 1.150 to 3.413, *P* = 0.014, with two studies estimated on the left side).

### Wound Complications

Five studies involving 55,092 patients reported on the rate of wound dehiscence, and the meta‐analysis showed that IDDM was associated with wound dehiscence (*RR*, 2.209, 95% *CI*, 1.596 to 3.040; *I* ^2^ = 38.7%). Surgical site infection (SSI) was reported separately in superficial SSI and deep incisional SSI with no heterogeneity (*I* ^2^ = 0%), with *RR* of 0.352 (95% *CI* 0.157 to 0.788) and 1.978 (95% *CI* 1.107 to 3.533), respectively.

### Infection

The most commonly reported complications in articles were sepsis, reoperation, and urinary tract infection. Seven studies including 79,398 patients reported the occurrence of sepsis for IDDM and NIDDM patients. After analysis of the results, which had an *I* ^2^ of 0%, the occurrence of sepsis was determined to be significantly higher in IDDM patients (*RR*, 1.965; 95% *CI*, 1.651 to 2.340). The pooled analysis also revealed that IDDM was associated with a high risk of urinary tract infection (*RR*, 1.407; 95% *CI* 1.214 to 1.631; *I* ^2^ = 40.3%). No significant differences were found in the occurrence of organ/space SSI.

### Other Complications

The extended length of stay (>5 days) that occurred during hospitality was documented in only 3 studies. The absence of heterogeneity among these articles allowed pooling of results and a higher risk of extended length of stay (*RR*, 1.509; 95% *CI*, 1.4109 to 1.616) was found in IDDM patients compared with NIDDM patients. Seven studies including 63,132 patients reported on the risk of reoperation. The pooled analysis showed that IDDM was associated with a higher risk of reoperation (*RR*, 1.460; 95% *CI*, 1.295 to 1.646) with no heterogeneity (*I* ^2^ = 0%). Seven studies involving 62,172 patients examined the effect of IDDM on readmission, and a significant increase in the rate of readmission was observed (*RR*, 1.494; 95% *CI*, 1.381 to 1.615; *I* ^2^ = 15.8%). It is worth mentioning that there were 6 studies involving 62,803 participants reporting on the risk of death in IDDM patients after TJA with no heterogeneity (*I* ^2^ = 0%) and the pooled analysis showed a greater than twofold risk of death in IDDM patients (*RR*, 2.292; 95% *CI*, 1.568 to 3.349). No significant differences were found in the occurrence of thrombotic events (VTE/PE) and revision.

## Discussion

The global prevalence of diabetes is expected to rise from 6.4% to 7.7% between 2010 and 2030^4^; therefore, its prevalence in the TJA population might increase accordingly. DM leads to a chronic low‐level inflammatory state, accompanied by metabolic disturbance, immune decline, and other negative states. Existing published studies show that diabetic patients are more likely to develop perioperative complications than non‐diabetic patients, including wound infection^25^, deep prosthesis infection^25^, ^26^, and prothesis revision^32^.

Lovecchio *et al*.^22^ further stratified 42,339 THA and TKA diabetic patients included in the National Surgical Quality Improvement Program into NIDDM and IDDM groups. The results showed that the risk of 30‐day mortality and readmission were significantly increased in IDDM patients, which was consistent with our pooled analysis. A retrospective study by Lee *et al*.^14^indicated that insulin dependence was associated with septic shock after rTKA, leading to renal insufficiency and even renal failure. Therefore, the authors recommend strengthening blood sugar control in IDDM patients to reduce the risk of renal failure after rTKA. In addition, IDDM also increases the risk of blood transfusion after rTKA compared with non‐diabetes mellitus and NIDDM.

Therefore, DM is a heterogeneous disease with varying severity in the TJA population. This situation must be taken into consideration when comparing the outcomes of patients with diabetes. Oliva *et al*.^33^ showed that applying personalized postoperative management based on perioperative risk stratification could reduce the incidence of postoperative complications. With the development of medical economics, further stratification of diabetes based on insulin dependence is critical to take into consideration the rapid growth of the diabetic population.

This analysis involved 81,697 patients undergoing TJA. The results showed that IDDM patients had significantly higher risk of adverse events than NIDDM patients. Although NIDDM patients had higher risk of superficial wound infection, IDDM patients’ risk of stroke, sepsis, myocardial infarction, extended length of stay (>5 days), being on a ventilator >48 h, renal failure, superficial incisional SSI, deep incisional SSI, reoperation, readmission, wound dehiscence, urinary tract infection, renal insufficiency, unplanned intubation, pneumonia, and death increased significantly.

The incidence of deep incisional SSI infection in IDDM patients was almost double that in NIDDM patients. Therefore, the risk of deep incision SSI in patients with more severe or long‐term chronic diabetes after TJA might increase. In contrast, the incidence of superficial SSI in IDDM is significantly lower than that in NIDDM, which may be because insulin effectively controls blood sugar levels in peripheral blood.

This is the first review, to our knowledge, to consider the effect of insulin on the complication rate of TJA, and this pooled analysis supports that clinicians should use the diabetic control method as a variable for risks in the preoperative evaluation of total joint replacement in diabetic patients. Our findings can help clinicians to stratify perioperative risks more accurately when questioning patients and provide a theoretical basis for adjusting perioperative management and setting postoperative expectations.

This meta‐analysis has several limitations. First, IDDM patients might be in the late stage of DM, during which pancreatic β cells are no longer functioning due to excessive cell damage. NIDDM patients may be in the early and middle stages of DM when pancreatic β cells are still functioning^34^. Second, we cannot further stratify the patients according to the level of HbA1c; that is, to evaluate insulin's control of HbA1c. The increased risk of complications in IDDM patients may be the result of an increased level of HbA1c. Finally, although the results of this study suggest that IDDM is a risk factor for increased incidence of complications after surgery, the study was unable to establish a causal link between IDDM and complications after surgery.

## Conclusion

Insulin dependence is a high‐risk factor for increased postoperative complications of TJA, and hierarchical and optimal blood glucose management may contribute to reducing the adverse complications after surgery. In addition, because the risk of sepsis, deep wound infection, organ/space SSI, urinary tract infection, renal insufficiency, and renal failure increased significantly after TJA in IDDM patients, more active postoperative antimicrobial prophylaxis may be needed on the premise of protecting renal function.

## Supporting information


**Appendix S1.** Grade evidence level of included studies

**Appendix S2.** PRISMA 2009 Checklist
