# Supplementary material for: Insulin Dependence Increases the Risk of Complications and Death in Total Joint Arthroplasty: A Systematic Review and Meta‐(Regression) Analysis
Source: Orthop Surg. 2021 Mar 18;13(3):719–33. doi: 10.1111/os.12944 (PMC8126943; doi:10.1111/os.12944)
Supplement: Supplementary file 1 — Appendix S1. Grade evidence level of included studies [file OS-13-719-s001.docx]

**Appendix 1.** Grade evidence level of included studies

| Outcomes | Quality Assessment | | | | | | | No. of Patients | | Effect | Quality | Importance |
| --- | --- | --- | --- | --- | --- | --- | --- | --- | --- | --- | --- | --- |
|  | No. of Studies | Design | Risk  of Bias | Inconsistency | Indirectness | Imprecision | Other Factors | IDDM | NIDDM | Risk Ratios  (95%CI) |  |  |
| Cardiac arrest | 5 | OB | None | Low | Low | Low | Large effect | 15,380 | 46,437 | 2.343  (1.546-3.550) | ⊕⊕⊕□ | Critical |
| Stroke | 6 | OB | Low | Low | Low | Low | Large effect | 15,675 | 47,128 | 2.180  (1.434- 3.313) | ⊕⊕⊕□ | Critical |
| Sepsis | 7 | OB | None | Low | Low | Low | None | 19,973 | 59,425 | 1.952  (1.643-2.319) | ⊕⊕□□ | Important |
| Myocardial infarction | 7 | OB | None | Low | Low | Low | None | 16,262 | 48,460 | 1.854  (1.442-2.382) | ⊕⊕□□ | Important |
| Extended length of stay(＞5 days) | 3 | OB | None | Low | Low | Low | None | 12,853 | 39,374 | 1.448  (1.362-1.539) | ⊕⊕□□ | Important |
| On ventilator ＞48h | 4 | OB | None | Low | Low | Low | Large effect | 15,380 | 46,437 | 2.918  (1.912-4.453) | ⊕⊕⊕□ | Critical |
| Renal failure | 5 | OB | None | Low | Low | Low | Large effect | 14,208 | 42,186 | 2.753  (1.822-4.158) | ⊕⊕⊕□ | Critical |
| Superficial incisional SSI | 2 | OB | None | Low | Low | Low | Large effect | 2,146 | 4,225 | 0.354  (0.159-0.790) | ⊕⊕⊕□ | Critical |
| Deep incisional SSI | 2 | OB | None | Low | Low | Low | None | 2,146 | 4,225 | 1.967  (1.106-3.498) | ⊕⊕□□ | Important |
| Organ/Space SSI | 2 | OB | None | Low | Low | Low | None | 2,146 | 4,225 | 0.916  (0.608-1.380) | ⊕⊕□□ | Important |
| Thrombotic event (VTE/PE) | 5 | OB | None | Low | Low | Low | None | 14,123 | 41,955 | 0.975  (0.829-1.148) | ⊕⊕□□ | Important |
| Reoperation | 7 | OB | Low | Low | Low | Low | None | 15,839 | 47,494 | 1.444  (1.286-1.622) | ⊕⊕□□ | Important |
| Readmission | 7 | OB | Low | Low | Low | Low | None | 15,122 | 47,050 | 1.494  (1.381-1.615) | ⊕⊕□□ | Important |
| Wound dehiscence | 4 | OB | Medium | Low | Low | Low | Large effect | 13,828 | 41,264 | 2.203  (1.596-3.040) | ⊕⊕⊕□ | Critical |
| Urinary tract infection | 7 | OB | Medium | Low | Low | Low | None | 15,793 | 47,339 | 1.400  (1.211-1.619) | ⊕⊕□□ | Important |
| Renal insufficiency | 5 | OB | Medium | Low | Low | Low | None | 14,123 | 41,955 | 1.971  (1.141-3.403) | ⊕□□□ | Important |
| Revision | 3 | OB | Medium | Low | Low | Low | None | 291 | 621 | 2.330  (0.681-7.974) | ⊕□□□ | Important |
| Unplanned intubation | 4 | OB | Medium | Low | Low | Low | None | 14,405 | 44,547 | 1.043  (0.715-1.521) | ⊕□□□ | Important |
| Pneumonia | 5 | OB | Medium | Low | Low | Low | None | 15,760 | 47,359 | 1.965  (1.238-3.120) | ⊕□□□ | Important |
| Death | 6 | OB | None | Low | Low | Low | Large effect | 15,675 | 47,128 | 2.292  (1.568-3.349) | ⊕⊕⊕□ | Critical |

**Abbreviations:** OB, Observation study; IDDM, Insulin-dependent diabetes mellitus; NIDDM, Non-insulin dependent diabetes mellitus.
